# Evaluation of Extraradicular Diffusion of Hydrogen Peroxide during Intracoronal Bleaching Using Different Bleaching Agents

**DOI:** 10.1155/2015/493795

**Published:** 2015-07-14

**Authors:** Mohammad E. Rokaya, Khaled Beshr, Abeer Hashem Mahram, Samah Samir Pedir, Kusai Baroudi

**Affiliations:** ^1^Department of Endodontics, Faculty of Dentistry, Al-Azhar University, Assiut, Egypt; ^2^Department of Restorative Dental Sciences, Al-Farabi Colleges, Riyadh, Saudi Arabia; ^3^Department of Endodontics, Faculty of Dentistry, Ain Shams University, Cairo, Egypt; ^4^Department of Preventive Dental Sciences, Al-Farabi Colleges, Riyadh, Saudi Arabia

## Abstract

*Objectives*. Extra radicular diffusion of hydrogen peroxide associated with intracoronal teeth bleaching was evaluated. *Methods*. 108 intact single rooted extracted mandibular first premolars teeth were selected. The teeth were instrumented with WaveOne system and obturated with gutta percha and divided into four groups (*n* = 27) according to the bleaching materials used. Each main group was divided into three subgroups (*n* = 9) according to the time of extra radicular hydrogen peroxide diffusion measurements at 1, 7, and 14 days: group 1 (35% hydrogen peroxide), group 2 (35% carbamide peroxide), group 3 (sodium perborate-30% hydrogen peroxide mixture), and group 4 (sodium perborate-water mixture). Four cemental dentinal defects were prepared just below the CEJ on each root surface. The amount of hydrogen peroxide that leached out was evaluated after 1, 7, and 14 days by spectrophotometer analysis. The results were analyzed using the ANOVA and Tukey's test. *Results*. Group 1 showed highest extra radicular diffusion, followed by group 3 and group 2, while group 4 showed the lowest mean extra radicular diffusion. *Conclusion*. Carbamide peroxide and sodium perborate-water mixture are the most suitable bleaching materials used for internal bleaching due to their low extra radicular diffusion of hydrogen peroxide.

## 1. Introduction

In modern society, greater emphasis is being placed on the cosmetic appearance of teeth [1]. Tooth discoloration is an aesthetic problem that may require treatment based on bleaching [2]. The causes of tooth discoloration are varied and complex but are usually classified as being either intrinsic or extrinsic in nature [3].

The main intrinsic factors are pulp hemorrhage, decomposition of pulp, bacteria and their products, tetracycline, pulp necrosis, intracanal medicaments, some endodontic filling materials, and metallic restorations, With the dissemination of blood components into the dentinal tubules caused by pulp extirpation or traumatically induced internal pulp bleeding, blood vessels are broken, promoting blood overflow into the pulp chamber [4]. Bleaching is considered as a procedure, which involves lightening the color of a tooth through application of a chemical agent to oxidize the organic pigmentation in the tooth [5].

Correct diagnosis of the cause of tooth discolouration is important, as shade changes as a result of different etiologies may require different treatment strategies. Thus, teeth with healthy pulps may be bleached by the home technique, termed night guard vital bleaching, or vital tooth bleaching, in-office techniques, or an association of both. Root filled teeth may be treated by the walking bleach technique, thermocatalytic technique, or an association of techniques [6].

Inside outside bleaching at home using carbamide peroxide in bleaching gel is safe but there are chances of collection of debris and contamination in the open pulp chamber when tray is not in mouth. In-office laser bleaching is a dentist controlled bleaching and is safe but may cost the patient quite a bit and one needs to buy specific equipment for the same. Walking bleach procedure, on the other hand, can be undertaken in remotest places also as no special equipment is required and material cost is also minimal [7].

The walking bleach technique involves the application of a bleaching agent to the dentine of the pulp chamber between dental visits. A mixture of sodium perborate and distilled water has been extensively used as an effective agent for intracoronal bleaching. In order to enhance bleaching efficacy, 30% hydrogen peroxide (HP) was suggested as a substitute for water. A concentrated hydrogen peroxide (25–35%) is efficient for bleaching teeth with or without vital pulp. Another bleaching agent, 35–37% carbamide peroxide (CP), has emerged as a popular and effective agent for both in-office and intracoronal bleaching techniques [8]. One of the most important properties of a bleaching agent is its ability to allow penetration of the bleaching agent through the dentinal permeability; the deeper the penetration is, the more the pigment that causes chromatic alteration of the dental tissue can be reversed by the oxidation reaction [9].

However, laboratory studies have demonstrated that intracoronal hydrogen peroxide can diffuse through the root, and this diffusion is greater in the presence of cemental root defects. Animal studies have confirmed the association of intracoronal bleaching with cervical root resorption. There is speculation that diffusion of hydrogen ions from intracoronal bleaching agents may provide an acidic environment that is optimal for osteoclastic activity and bone resorption, resulting in external cervical root resorption over time [10].

Since dentinal tubules are oriented incisally, placing a protective base material at the lower level of the CEJ could reduce the leakage of hydrogen peroxide to the periodontal tissues. Deferent protective base materials have been compared. No significant differences were found between them [11].

This study aimed to compare by spectrophotometer analysis using potassium permanganate the extraradicular diffusion of hydrogen peroxide associated with intracoronal teeth bleaching by Opalescence Endo 35%, Opalescence PF 35%, sodium perborate with 30% hydrogen peroxide mixture, and sodium perborate with water mixture.

## 2. Materials and Methods

108 freshly extracted human single root canals (mandibular first premolars) for orthodontic reasons from patient less than 21 years old were collected with no sign of cracks or structural anomalies. A prior patient's consent was given to use their extracted teeth to conduct the study. Approval of Al-Azhar University Faculty of Oral and Dental Medicine, Egypt (under number 282/2009), was also obtained. The selected teeth were then immersed in 5.25% sodium hypochlorite for one hour to dissolve organic debris that was present on the external surface of the roots. Subsequently, they were cleaned with an ultrasonic scaler (satelec, Acteon, France) to remove calculus, discarding teeth with previous root canal treatment, internal resorption, and external resorption, localized or diffuse calcifications. The selected teeth were stored in normal saline at room temperature until the time of use.

A standardized access cavity was made using a carbide bur loaded in a high speed handpiece. Each root canal orifice was enlarged using Gates-Glidden bur numbers 3 and 4 to enlarge root canals of the same size using 5.25% NaOCl solution for irrigation. The pulp tissues were removed with a barbed broach (Dentsply Maillefer, Ballaigues, Switzerland). Working length (WL) was determined by inserting a size 10 K-file (Dentsply Maillefer, Ballaigues, Switzerland) which was passively introduced into the canal until the tip was seen to exit at the major foramen. The real length of the canal was recorded and the working length calculated by subtracting 1 mm from this measurement.

The preparation was performed with a single rotary file, the WaveOne (Dentsply Maillefer, Ballaigues, Switzerland), and the preprogrammed motor (X-Smart Plus, Dentsply Maillefer, Ballaigues, Switzerland), using a specific movement of reciprocation: 170°CCW and 50°CW at a speed equivalent to 350 rpm.

Root canals were initially shaped with WaveOne primary reciprocating file (25/08) in the presence of 5.25% NaOCl as irrigant. The primary WaveOne file was used gently in a reciprocating, slow in- and out-pecking motion with short 2-3 mm amplitude strokes, to passively advance until it does not easily progress anymore. The shaping step was repeated until the coronal two-thirds of the canal has been prepared. The working length and canal patency were confirmed and then the primary file is carried to the full working length in one or more passes.

A larger WaveOne file 40/08 was carried to working length to fully shape and finish the preparation. The finished shape is confirmed in which the apical flutes of the file are loaded with dentin and a gauging 40/02 hand file is snugged at length. The apical foramen of each root was sealed with wax during instrumentation to prevent irrigating solutions from passing through the apical foramen. During instrumentation, the canals were irrigated with 2 mL of 5.25% NaOCl solution using a plastic syringe with 30-gauge closed-end needle (Hawe Max-I-probe, Hawe Neos, Bioggio, Switzerland). The needle was inserted within 1 to 2 mm of the working length. Then the root canals were dried by size 40/08 WaveOne paper point. Root canals are obturated with size 40/08 WaveOne gutta-percha using ProRoot Endo Sealer (Dentsply Maillefer, Ballaigues, Switzerland).

**Figure 1:**
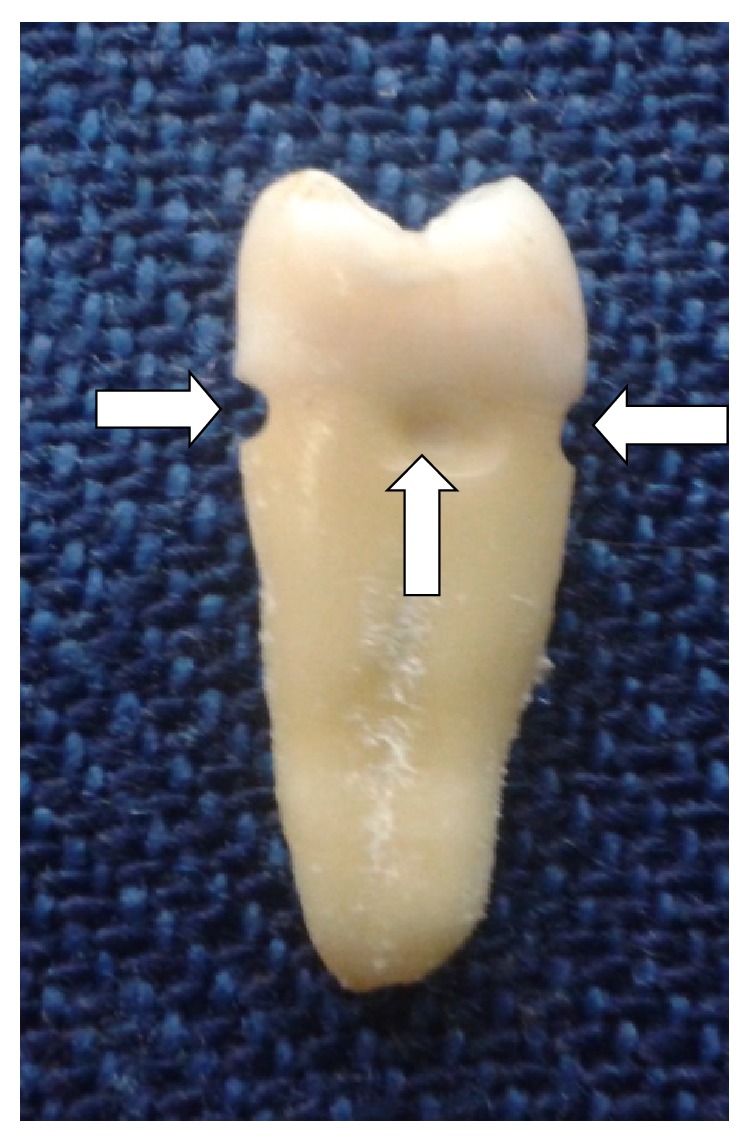
A photograph showing simulated defects below CEJ.

To simulate standardized breaks in the cemental covering, four cemental dentinal defects were prepared just below the CEJ on each root surface, mesial, distal, buccal, and lingual aspects [10]. The hemispherical defects (diameter 1.0 mm, depth 0.5 mm) were created using a round diamond bur (diameter 1.0 mm) in a high speed handpiece. The smear layer created in the defects was removed with 15% EDTA and then thoroughly washed with distilled water. The apical two-thirds of the root surface was coated with a double layer of a nail varnish to seal potential superficial defects as in Figure 1.

### 2.1. Specimen's Grouping

The prepared specimens were then divided randomly into four groups (*n* = 27) according to the beaching materials used. Each main group was subdivided into three subgroups (*n* = 9) according to the time of extraradicular hydrogen peroxide diffusion measurements at 1, 7, and 14 days.

#### 2.1.1. Group 1 (Opalescence Endo 35%)

Opalescence Endo (Ultradent Products, South Jordan, UT, USA) is a 35% hydrogen peroxide specially formulated gel for the “walking” bleach technique (pH 5).

#### 2.1.2. Group 2 (Opalescence PF 35%)

Opalescence PF 35% (Ultradent Products, South Jordan, UT, USA) is 35% carbamide peroxide, 0.5% potassium nitrate, and 0.11% weight to weight (1100 ppm) fluoride ion. It is clear, high-viscosity, and sticky (pH 6.5).

#### 2.1.3. Group 3 (Sodium Perborate-30% Hydrogen Peroxide Mixture)

There is a mixture of sodium perborate (Degussa, Hanau, Germany) and 30% hydrogen peroxide (in the ratio, 1 g of powder : 0.5 mL of liquid) (pH 7.1).

#### 2.1.4. Group 4 (Sodium Perborate-Water Mixture)

There is a mixture of sodium perborate (Degussa, Hanau, Germany) and distilled water (in the ratio, 1 g of powder: 0.5 mL of distilled water) (pH 9.7).

### 2.2. Bleaching Procedure

The root filling in the coronal pulp chamber was removed to 2 mm below the facial cementoenamel junction. A barrier placement of 2 mm layer of glass-ionomer cement (Ketac-Molar, 3 M ESPE, St. Paul, MN, USA) was placed over the gutta-percha [12]. A pellet of cotton was placed in the chamber; it was sealed temporarily with Cavit (3 M ESPE, St. Paul, MN, USA). After 24 hours, the temporary filling and the cotton pellet were removed completely.

The walls of the access cavity were cleaned of any residue using a small carbide bur followed by thorough rinsing with 2 mL of 5.25% NaOCl. The bleaching material of each group was placed in the pulp chamber and the access cavity of each specimen sealed temporarily with Cavit.

Each tooth was suspended in a plastic vial of 3 mL distilled water using laboratory sealing film Parafilm (Parafilm M, Neenah, WI, USA). The sealing film was cut to fit each tooth at the level of the CEJ and stabilized with sticky wax to achieve a tight seal and stored in an incubator at 37°C and 100% relative humidity during 14 days of bleaching; the bleaching materials were changed at 7 days, and the amount of hydrogen peroxide that leached out into the distilled water was evaluated after 1, 7, and 14 days.

## 3. Extraradicular Hydrogen Peroxide Diffusion Measurement

In this study we used titration method of permanganate and hydrogen peroxide according to the following equation [13]

(1) $$\begin{array}{c} 2Mn{O_{4}}^{-}+5H_{2}{O_{2}}^{+}+6H^{+}⟶2{{Mn}_{2}}^{+}+5O_{2}+8H_{2}O \end{array}$$

**Figure 2:**
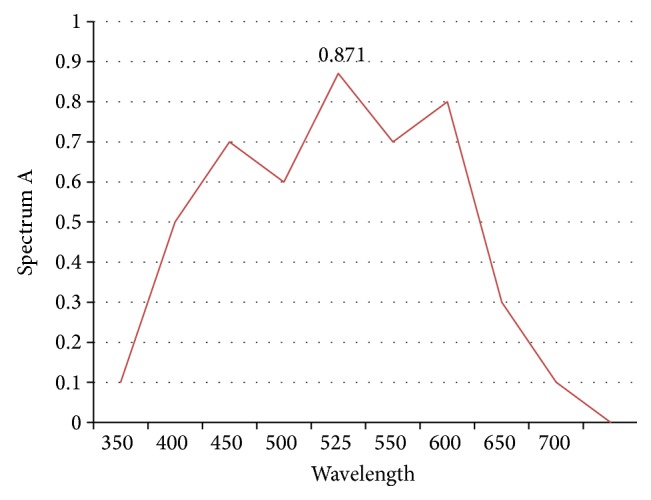
A schematic diagram showing KMnO_4_ having a standard spectrum (absorbance 0.871 A) at wavelength 525 nm.

The pink color of permanganate would be changed when reacted with peroxides; the color disappears according to the concentration of peroxide. The reaction is fast and does not need time or temperature for progression. The amount of radicular peroxide was measured using the UV-visible spectrophotometer (SpectraMax 1601, Molecular Devices Corp., Sunnyvale, CA, USA) at a wavelength of 480 nm (at room temperature), utilizing the potassium permanganate (KMnO_4_) as peroxide indicator (redox reaction) and (KMnO_4_) having a standard spectrum (absorbance 0.871 A) at wavelength 525 nm (Figure 2).

## 4. Results

### 4.1. After 1 Day

Group 1 (Opalescence Endo 35%) showed the statistically significantly highest extraradicular diffusion (0.168 ± 0.049) *P* < 0.05. There was no statistically significant difference between Group 2 (Opalescence PF 35%), Group 3 (sodium perborate-30% hydrogen peroxide mixture), and Group 4 (sodium perborate-water mixture) which showed the statistically significantly lowest mean extraradicular diffusion (Groups 2 and 4 showed no extraradicular diffusion).

### 4.2. After 7 Days

Group 1 (Opalescence Endo 35%) showed highest extraradicular diffusion (0.482 ± 0.051). This was followed by Group 3 (sodium perborate-30% hydrogen peroxide mixture (0.375 ± 0.049)) and Group 2 (Opalescence PF 35% (0.210 ± 0.125)) while Group 4 (sodium perborate-water mixture) showed the lowest mean extraradicular diffusion (0.141 ± 0.040). Statistically there was no statistically significant difference between Group 1 and Group 3 while there was statistically significant difference between the other tested groups. Meanwhile there was no statistically significant difference between Groups 2 and 4.

### 4.3. After 14 Days

Group 1 (Opalescence Endo 35%) showed highest extraradicular diffusion (0.865 ± 0.061). This was followed by Group 3 (sodium perborate-30% hydrogen peroxide mixture (0.743 ± 0.060)) and Group 2 (Opalescence PF 35% (0.430 ± 0.30)) while Group 4 (sodium perborate-water mixture) showed the lowest mean extraradicular diffusion (0.315 ± 0.046). Statistically there was no statistically significant difference between Group 1 and Group 3 while there was statistically significant difference between the other tested groups. Meanwhile there was no statistically significant difference between Groups 2 and 4.

### 4.4. On the Other Hand

All bleaching material used in this study recorded statistically significant increase in mean extraradicular diffusion after 7 and 14 days (Table 1).

## 5. Discussion

The current study employed the use of four different bleaching agents with intracoronal teeth bleaching and they are 35% hydrogen peroxide (Opalescence Endo), 35% carbamide peroxide (Opalescence PF 35%), sodium perborate with 30% hydrogen peroxide mixture, and sodium perborate with water mixture.

This study has been done to investigate the effect of these four bleaching agents on extraradicular hydrogen peroxide diffusion after one and seven days and fourteen days by potassium permanganate (KMnO_4_) as peroxide indicator. Understanding the chemistry of the active ingredient of the bleaching agent would be useful.

Hydrogen peroxide (H_2_O_2_) is the main whitening agent employed. It is a thermally unstable free radical with a low molecular weight, which penetrates the enamel and dentin through diffusion. Complex molecules of organic pigments in the tissues are broken down into simpler hydrophilic molecules through an oxidation reduction reaction by the action of perhydroxyl ions originating from the degradation of H_2_O_2_. These simpler molecules are easily removed from the dental tissue when being in contact with water, thereby providing the desired whitening effect [14, 15].

Carbamide peroxide (CH_4_N_2_O·H_2_O_2_) decomposes to 1/3 hydrogen peroxide and 2/3 urea [16]. The urea can theoretically be further decomposed to carbon dioxide and ammonia, which elevates the pH to facilitate the bleaching procedure further. This can be explained by the fact that, in a basic solution, lower activation energy is required for the formation of free radicals from hydrogen peroxide, and the reaction rate is higher [17].

Sodium perborate is an oxidizing agent available as a powder. It is stable when being dry; however, in the presence of acid, warm air, or water, it breaks down to form sodium metaborate, hydrogen peroxide, and nascent oxygen. Sodium perborate is easier to control and safer than concentrated hydrogen peroxide solutions [18].

This study compared extraradicular hydrogen peroxide diffusion of various bleaching agents after one and seven days and fourteen days. Previous studies of peroxide penetration have utilized different time periods [11, 19].

In the present study, single rooted mandibular premolars extracted for orthodontic reasons from patients below 21 years were used. They were selected because of the availability of such teeth and most reported cases of cervical root resorption associated with intracoronal bleaching were in young patients. It is probable that the wide and patent dentinal tubules in young teeth would favor ionic diffusion of the bleaching agent through dentine [10].

The walking bleach technique requires a sound coronal seal around the access cavity after application of bleaching paste in the chamber. It can be achieved with GIC, resin composite, or compomer to ensure its effectiveness and to avoid leakage of bleaching agent into the oral cavity. In addition, a good seal also prevents recontamination of the dentin with microorganisms and reduces the risk of renewed staining [18].

In the present study, although the teeth were not totally immersed in the distilled water, the access openings were protected in order to prevent the leakage of bleaching agents through this area. Thus, the diffusion of the bleaching material occurred only through the cervical dentin tubules, exposed by CJE defects. It is important to emphasize the difficulty to obtain teeth with similar CJE characteristics, which lead some researchers to create artificial defects along the external cervical area to standardize the diffusion of bleaching agents [20].

In this study, a 2 mm layer of glass-ionomer cement was applied over the canal filling due to its effectiveness in preventing penetration of 30% hydrogen peroxide solution into the root canal. Thus, the use of this material as a base during bleaching presents the additional advantage that it can be left in place after bleaching and can serve as a base for the final restoration [12].

*The result of this study showed that* after 1 day, Group 1 (*Opalescence Endo 35%*) showed the statistically significantly highest extraradicular diffusion *P* < 0.05. There was no statistically significant difference between group 2 (Opalescence PF 35%), group 3 (sodium perborate-30% hydrogen peroxide mixture), and group 4 (sodium perborate-water mixture) after 7 and 14 days. Group 1 showed highest extraradicular diffusion, followed by group 3 and group 2, while group 4 showed the lowest mean extraradicular diffusion.

*The probable reasons for the results* obtained in the present study could be [21] as follows. (a) As 35% carbamide peroxide decomposes on contact with moisture to yield approximately only 12% hydrogen peroxide. (b) It could be because of the fact that carbamide peroxide does not diffuse through dentine as fast as hydrogen peroxide. (c) With the rise in pH aided by the resultant ammonia from the carbamide peroxide, the deionization of the hydrogen peroxide is facilitated. Therefore, little unreacted hydrogen peroxide is left to diffuse through the root dentin into the extraradicular environment. These results were in agreement with that of Gökay et al. [22] who stated that higher peroxide penetration occurred with the 30% HP-SP mixture than with the CP bleaching gels, and the 37% CP group also promoted greater peroxide penetration than the other CP groups (*P* < 0.05). There was no statistically significant difference between 10% and 17% CP groups (*P* > 0.05). Lee et al. [10] stated that carbamide peroxide had very low levels of extraradicular diffusion of HP. Shivanna and Gupta [21] concluded that radicular peroxide penetration from carbamide peroxide gels is less than sodium perborate-hydrogen peroxide mixture, therefore carrying less risk of postbleaching external root resorption.

Heithersay analyzed cervical resorption cases and reported that 24.1% were caused by orthodontic treatment, 15.1% by dental trauma, 5.1% by surgery (e.g., transplantation or periodontal surgery), and 3.9% by intracoronal bleaching. A combination of internal bleaching with one of the other causes is responsible for 13.6% of cervical resorption cases. The combination of bleaching and history of trauma is the most important predisposing factor for cervical resorption [23].

## 6. Conclusion

Carbamide peroxide and sodium perborate-water mixture are the most suitable bleaching materials used for internal bleaching due to its low extraradicular diffusion of hydrogen peroxide.

## Figures and Tables

**Table 1:** The means, standard deviation (SD) values, and results of ANOVA and Tukey's test for comparison of extraradicular diffusion between four groups.

Time	Group 1		Group 2		Group 3		Group 4		*P* value
	(Opalescence Endo 35%)		(Opalescence PF 35%)		(sodium perborate-30% hydrogen peroxide mixture)		(sodium perborate-water mixture)		
	Mean	S.D	Mean	S.D	Mean	S.D	Mean	S.D	
1 day	0.168^c^	0.049	0^d^	0	0.068^d^	0.021	0^d^	0	^*∗*^ < 0.001
7 days	0.482^b^	0.051	0.210^c^	0.125	0.375^b^	0.049	0.141^c^	0.040	
14 days	0.865^a^	0.061	0.430^b^	0.030	0.743^a^	0.060	0.315^b^	0.046	

^*∗*^Significant at *P* ≤ 0.05; means with different letters are statistically significantly different according to Tukey's test.

## References

[1] de Souza-Zaroni W. C., Lopes E. B., Ciccone-Nogueira J. C., Silva R. C. S. P. (2009). Clinical comparison between the bleaching efficacy of 37% peroxide carbamide gel mixed with sodium perborate with established intracoronal bleaching agent. *Oral Surgery, Oral Medicine, Oral Pathology, Oral Radiology, and Endodontology*.

[2] Kaneko J., Inoue S., Kawakami S., Sano H. (2000). Bleaching effect of sodium percarbonate on discolored pulpless teeth in vitro. *Journal of Endodontics*.

[3] Addy M., Moran J., Newcombe R., Warren P. (1995). The comparative tea staining potential of phenolic, chlorhexidine and anti-adhesive mouthrinses. *Journal of Clinical Periodontology*.

[4] Valera M. C., Camargo C. H. R., Carvalho C. A. T., de Oliveira L. D., Camargo S. E. A., Rodrigues C. M. (2009). Effectiveness of carbamide peroxide and sodium perborate in non-vital discolored teeth. *Journal of Applied Oral Science*.

[5] Feiz A., Barekatain B., Khalesi S., Khalighinejad N., Badrian H., Swift E. J. (2014). Effect of several bleaching agents on teeth stained with a resin-based sealer. *International Endodontic Journal*.

[6] Yui K. C. K., Rodrigues J. R., Mancini M. N. G., Balducci I., Gonçalves S. E. P. (2008). Ex vivo evaluation of the effectiveness of bleaching agents on the shade alteration of blood-stained teeth. *International Endodontic Journal*.

[7] Anuradha R., Manisha G. (2009). Walking bleach-still relevant; a review with—a case report. *Indian Journal of Dental Sciences*.

[8] Cavalli V., Shinohara M. S., Ambrose W., Malafaia F. M., Pereira P. N. R., Giannini M. (2009). Influence of intracoronal bleaching agents on the ultimate strength and ultrastructure morphology of dentine. *International Endodontic Journal*.

[9] Carrasco L. D., Fröner I. C., Corona S. A. M., Pécora J. D. (2003). Effect of internal bleaching agents on dentinal permeability of non-vital teeth: quantitative assessment. *Dental Traumatology*.

[10] Lee G. P., Lee M. Y., Lum S. O. Y., Poh R. S. C., Lim K.-C. (2004). Extraradicular diffusion of hydrogen peroxide and pH changes associated with intracoronal bleaching of discoloured teeth using different bleaching agents. *International Endodontic Journal*.

[11] Lambrianidis T., Kapalas A., Mazinis M. (2002). Effect of calcium hydroxide as a supplementary barrier in the radicular penetration of hydrogen peroxide during intracoronal bleaching in vitro. *International Endodontic Journal*.

[12] Rotstein I., Zyskind D., Lewinstein I., Bamberger N. (1992). Effect of different protective base materials on hydrogen peroxide leakage during intracoronal bleaching in vitro. *Journal of Endodontics*.

[13] Campanella L., Roversi R., Sammartino M. P., Tomassetti M. (1998). Hydrogen peroxide determination in pharmaceutical formulations and cosmetics using a new catalase biosensor. *Journal of Pharmaceutical and Biomedical Analysis*.

[14] Pinto M., de Godoy C. H., Bortoletto C. (2014). Tooth whitening with hydrogen peroxide in adolescents: study protocol for a randomized controlled trial. *Trials*.

[15] Pinto M. M., Bussadori S. K., Guedes-Pinto A. C., Rego M. A., Eberson P. (2004). Esthetic alternative for fluorosis blemishes with the usage of a dual bleaching system based on hydrogen peroxide at 35%. *Journal of Clinical Pediatric Dentistry*.

[16] Baltzer A., Kaufmann-Jinoian V. (2005). Shading of ceramic crowns using digital tooth shade matching devices. *International Journal of Computerized Dentistry*.

[17] Heithersay G. S., Dahlstrom S. W., Marin P. D. (1994). Incidence of invasive cervical resorption in bleached root-filled teeth. *Australian Dental Journal*.

[18] Plotino G., Buono L., Grande N. M., Pameijer C. H., Somma F. (2008). Nonvital tooth bleaching: a review of the literature and clinical procedures. *Journal of Endodontics*.

[19] Benetti A. R., Valera M. C., Mancini M. N. G., Miranda C. B., Balducci I. (2004). In vitro penetration of bleaching agents into the pulp chamber. *International Endodontic Journal*.

[20] Rotstein I., Torek Y., Misgav R. (1991). Effect of cementum defects on radicular penetration of 30% H_2_O_2_ during intracoronal bleaching. *Journal of Endodontics*.

[21] Shivanna V., Gupta K. (2012). A spectrophotometric analysis of radicular peroxide penetration after intracoronal bleaching using different concentrations of carbamide peroxide gels. *Endodontology*.

[22] Gökay O., Ziraman F., Asal A. Ç., Saka O. M. (2008). Radicular peroxide penetration from carbamide peroxide gels during intracoronal bleaching. *International Endodontic Journal*.

[23] Heithersay G. S. (1999). Invasive cervical resorption following trauma. *Australian Endodontic Journal*.

